# Boosting Electro- and Photo-Catalytic Activities in Atomically Thin Nanomaterials by Heterointerface Engineering

**DOI:** 10.3390/ma16175829

**Published:** 2023-08-25

**Authors:** Xingyu Chen, Xinyue Jiang, Hao Zhang

**Affiliations:** 1 School of Metallurgical and Ecological Engineering, University of Science and Technology Beijing, Beijing 100083, China; 2 School of Energy and Environmental Engineering, University of Science and Technology Beijing, Beijing 100083, China

**Keywords:** atomically thin materials, heterointerface engineering, electronic structure, electrocatalysis, energy storage, energy conversion

## Abstract

Since the discovery of graphene, two-dimensional ultrathin nanomaterials with an atomic thickness (typically <5 nm) have attracted tremendous interest due to their fascinating chemical and physical properties. These ultrathin nanomaterials, referred to as atomically thin materials (ATMs), possess inherent advantages such as a high specific area, highly exposed surface-active sites, efficient atom utilization, and unique electronic structures. While substantial efforts have been devoted to advancing ATMs through structural chemistry, the potential of heterointerface engineering to enhance their properties has not yet been fully recognized. Indeed, the introduction of bi- or multi-components to construct a heterointerface has emerged as a crucial strategy to overcome the limitations in property enhancement during ATM design. In this review, we aim to summarize the design principles of heterointerfacial ATMs, present general strategies for manipulating their interfacial structure and catalytic properties, and provide an overview of their application in energy conversion and storage, including the hydrogen evolution reaction (HER), the oxygen evolution reaction (OER), the oxygen reduction reaction (ORR), the CO_2_ electroreduction reaction (CO_2_RR), photocatalysis, and rechargeable batteries. The central theme of this review is to establish correlations among interfacial modulation, structural and electronic properties, and ATMs’ major applications. Finally, based on the current research progress, we propose future directions that remain unexplored in interfacial ATMs for enhancing their properties and introducing novel functionalities in practical applications.

## 1. Introduction

The prosperity of modern society highly relies on the consumption of fossil fuels, such as coal, petroleum, and natural gas [1,2,3]. However, the extensive exploitation of these resources not only accelerates their depletion but also causes severe environmental issues and health concerns [4,5]. In response to the energy crisis of this century, the pursuit of energy generation on the basis of renewable and environmentally friendly resources has been a primary concern [6,7,8]. As a result, the adoption of renewables, including wind, solar, and hydropower, in overall energy consumption is steadily rising, in line with the demand for high-energy-density fuels and their utilization and storage techniques [9,10,11,12]. Electrocatalysis has emerged as a crucial solution for various energy conversion processes, such as the hydrogen evolution reaction (HER) for hydrogen production as an alternative fuel, the CO_2_ electroreduction reaction (CO_2_RR) for high-value products and liquid fuels, oxygen catalysis (the oxygen reduction reaction (ORR)/the oxygen evolution reaction (OER)) for hydrogen fuel cells and rechargeable metal–air batteries, and photocatalysis for directly harvesting solar energy [13,14,15,16,17]. However, the performance of traditional catalysts falls short due to their sluggish kinetics, limited stability, and low selectivity [18,19,20]. Consequently, the development of advanced catalysts remains a prominent focus in both academic and industrial sectors.

Recently, atomically thin materials (ATMs), inspired by the discovery of graphene in 2004 [21], have opened up new avenues for designing next-generation advanced catalysts [22]. ATMs represent a new class of ultrathin two-dimensional (2D) nanomaterials, with a thickness of typically less than 5 nm. Their atomic-scale thickness imparts inherent advantages, such as (i) a large specific surface area with a shortened channel that facilitates a faster charge/reactant transfer, resulting in accelerated surface-donated and diffusion-controlled reaction kinetics; (ii) an extremely high exposure of inner atoms optimizing atom utilization to its largest extent; and (iii) a tunable electronic structure and exotic catalytic properties. These merits make ATMs an ideal 2D platform for chemical modulation and mechanical study at an atomic-thin scale [23,24]. Numerous strategies have been explored to optimize ATMs, including defect engineering (vacancy or chemical doping), alloying, the formation of secondary phases, a solid solution, chemical substitution, the controlled growth of active planes/edges, and heteroatom decoration.

Among the various strategies, heterointerfacial engineering has emerged as an outstanding approach to design advanced catalysts based on ATM composites. The introduction of alien phases to ATMs to construct heterostructures offers a broader space for material design and property enhancement. Firstly, from a chemical perspective, the heterointerfacial strategy directly integrates the alien phase and ATM’s matrix rather than putting alien components inside the matrix lattice, allowing greater flexibility in selecting alien components beyond the matrix lattice. Secondly, the heterointerface synergistically promotes the catalytic reaction by combining the merits of both phases. Their interaction with each other could modulate the electronic structure around the interface to benefit the catalytic process. Thirdly, the heterointerface provides multiple adsorption sites that may independently optimize the adsorption energy of different intermediates, facilitating the overall reaction. Lastly, the built-in electric field (BIEF) around the interface, arising from the electron redistribution between two adjacent phases due to their difference in Fermi levels, may optimize the adsorption of different reactants through the Coulomb interaction. Here, in this review, we discuss ATMs with a delicately designed heterointerface. First, we introduce the design principles of heterointerfacial ATMs, which are generally categorized as either 2D/2D heterostructures or 0D/2D heterostructures. Next, detailed interfacial strategies for developing advanced ATMs are widely discussed, encompassing the direct and indirect modulations of the electronic structure and charge/electron transfer, to establish a clear interfacial structure–property relationship. Subsequently, their applications in catalysis (i.e., HER, OER, ORR, and CO_2_RR), photocatalysis, and rechargeable batteries are summarized. Finally, we outline the challenges and opportunities in fabricating novel heterointerfacial ATMs for energy conversion and storage applications.

## 2. Design Principles for Heterointerfacial ATMs

ATMs inherently possess superiority in their morphology and structure compared to traditional nanomaterials, as their ultrathin thickness on an atomic scale endows these materials with a very high possibility of exposing inner atoms and, correspondingly, achieving ultrahigh atomic utilization [24]. However, to better suit energy storage and conversion devices, further optimization of their intrinsic electrocatalytic activity and electronic structure is necessary. Building a heterointerface in the catalyst allows for the easy tuning of the band structure around the interface, as long as the heterophase can bond with and interact with ATMs. Moreover, the choice of elements for the heterophase can be diverse, and the resulting heterostructure can effectively leverage the strength of both phases, leading to a superior performance compared to that of ATMs. As ATMs possess unsaturated surface dangling bonds, their electronic structure can be easily modulated through chemical doping or heterostructure construction. Additionally, the interface provides a channel for charge/electron transfer and a short pathway for mass transfer, thereby enhancing the overall redox ability and boosting the reaction kinetics [25]. Therefore, designing a heterointerface on ATMs represents a promising and feasible approach to expand the frontier of knowledge in electrocatalyst design. Herein, we classify the fundamental design principles of the heterointerfacial ATMs into two main categories based on the dimensions of the resultant catalysts, namely 2D/2D heterostructures and 0D/2D heterostructures. The most commonly discussed heterostructures are 2D/2D heterostructures, as their large lateral surface features ensure a substantial number of highly exposed surface atoms to actively participate in the reaction [26]. Furthermore, we also include 0D/2D heterostructures, referring to single atoms dispersed on 2D ATMs. The emergence of single atomically dispersed catalysts (SACs) represents a breakthrough in heterogeneous catalysts, as they require minimal noble metal loading to achieve outstanding catalytic performance, making them a resource-saving approach for highly efficient catalysis [27,28]. Consequently, the loading of single dispersed atoms on ATMs is also included in our discussion, and we treat them as a special “interface” in a broad sense.

### 2.1. Two-Dimensional/Two-Dimensional Heterostructures

**Figure 1:**
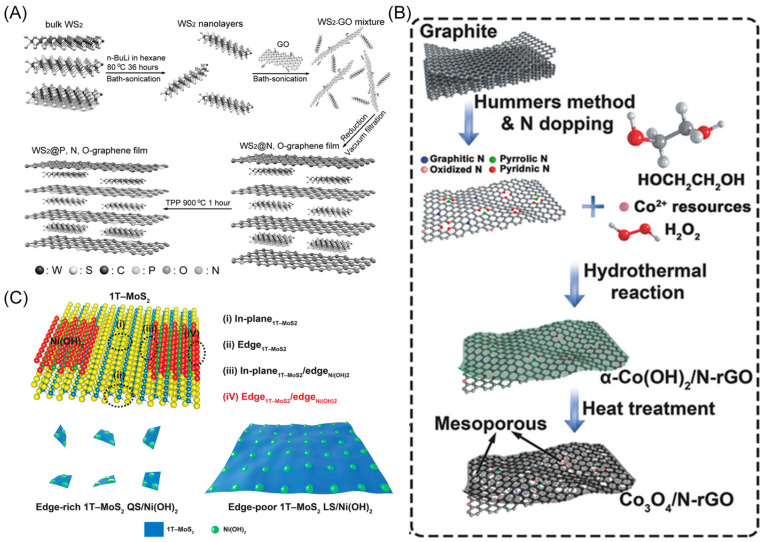
(**A**) Schematic illustration of the fabrication process of the WS_2_@P, N, O-graphene sheet. Reproduced with permission: Copyright 2015, Wiley-VCH [30]. (**B**) Schematic illustration of the synthesis process of the 2D Co_3_O_4_/N-rGO nanosheets for the knittable zinc–air batteries. Reproduced with permission: Copyright 2017, Wiley-VCH [31]. (**C**) Schematic illustration of four positions of active sites (upper), edge-rich 1T–MoS_2_ quantum sheets (QS)/Ni(OH)_2_ (bottom left) and edge-poor 1T–MoS_2_ large sheets (LS)/Ni(OH)_2_ (bottom right) catalysts. Reproduced with permission: Copyright 2020, Wiley-VCH [32].

Fabricating 2D/2D heterostructures with overlapping ATMs allows for the creation of catalysts with an overall atomic thickness, thus retaining and enhancing the merits of ATMs through the generated heterointerface. This special construction alters the electronic structure and Fermi level positions [29], leading to enhanced charge transfer ability and optimized adsorption/desorption energy of catalytic sites for reactants/intermediates [24]. In most cases, 2D/2D heterostructures are primarily constructed using van der Waals interactions and chemical bonds [23]. Graphene, known for its superior conductivity, is commonly used as a substrate. For instance, Qiao’s group fabricated a heterostructure by assembling 2D WS_2_ nanolayers with P-, N-, and O-doped graphene sheets (WS_2_@P, N, O-graphene film) via a vacuum filtration process [30]. The van der Waals forces ensure tight attachment of the heterostructure (Figure 1A). The resultant catalyst delivers a commercial Pt/C-comparable overpotential of 125 mV at a current density of 10 mA cm^−2^, with a low Tafel slope of 52.7 mV dec^−1^. Similarly, Lu et al. built a Co_3_O_4_/N-rGO heterostructure through hydrothermal reactions and heat treatment [31]. The strong coupling between Co_3_O_4_ and N-rGO boosts the electronic interaction (Figure 1B). As confirmed by the X-ray absorption near-edge structure (XANES) results, the electrons transfer from Co_3_O_4_ to N-rGO, altering the electronic structure, leading to enhanced oxygen-related intermediates’ adsorption/desorption processes and improving ORR and OER catalytic performance. As an ORR catalyst, it exhibits an onset potential of 0.92 V and a higher limiting current density of 5.34 mA cm^−2^, similar to those of a commercial Pt/C. Its unique flexible structure makes it applicable to knittable fiber-shaped zinc–air batteries. Transition metal dichalcogenides (TMDs) are used for the substrate due to their layered structure. Gu and co-workers fabricated Ni(OH)_2_-decorated 1T-MoS_2_ quantum sheets to deliver a highly active HER catalyst via interface engineering (Figure 1C) [32]. Through the density functional theory (DFT) calculation, the edge_1T-MoS2_/edge_Ni(OH)2_ heterostructures exhibit the most optimal H_2_O adsorption energy and moderate adsorption free energy of H and OH compared to the other three presumed active sites. This results in the most outstanding intrinsic HER activity, with a small overpotential of 112 mV to drive a high current density of 100 mA cm^−2^ and a low Tafel slope of 30 mV dec^−1^ in 1.0 M KOH. It also exhibits long durability for 100 h at a current density up to 500 mA cm^−2^.

### 2.2. Zero-Dimensional/Two-Dimensional Heterostructures

**Figure 2:**
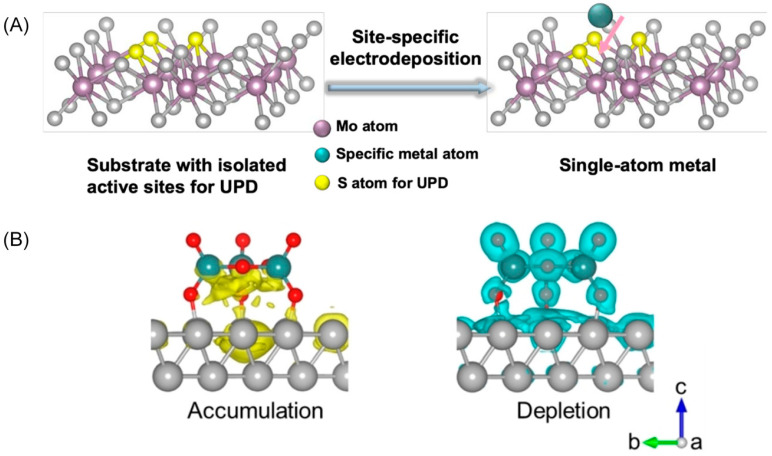
(**A**) Schematic illustration of the deposition of single-atom metal on the transition metal dichloride (MoS_2_) substrate. Reproduced with permission: Copyright 2020, Springer Nature [34]. (**B**) Schematic illustration of charge density difference at the interfaces of MoO*_x_*–Rh metallene. The electrons transfer and accumulate at the interface (the yellow area), leaving an electron depletion area (the green one) outside the interface. Reproduced with permission: Copyright 2022, Wiley-VCH [35].

Anchoring single-atom scale materials on ultrathin substrates, termed SAC-anchored ATMs, represents another effective and favorable material design approach for fabricating heterointerfacial atomically thin interface materials. Herein, we categorize these SAC-anchored ATMs as 0D/2D heterostructures due to the presence of a micro-interface between the single atom and the substrate. SACs drastically decrease noble metal usage while maintaining satisfactory catalytic activity. Combining SACs with atomically thin substrates further improves loading density, providing a resource-saving and effective method for novel catalyst exploitation [33]. For instance, Xia’s group developed a feasible room-temperature electrodeposition method for anchoring single-atom metals onto the chalcogen atoms of TMDs [34]. Driving the applied potential and the affinity of metal adatoms to chalcogen atoms on the substrate, the single-atom metal is thermodynamically favorably fixed on the supports. This process leads to the self-terminating growth of atomically dispersed metal, as illustrated in Figure 2A. The single Pt atom anchored on the MoS_2_ substrate enhances the intrinsic catalytic activity, delivering outstanding performance with a small overpotential of ca. 59 mV at 10 mA cm^−2^ and a low Tafel slope of ca. 31 mV dec^−1^. This methodology is versatile, extended to the synthesis of a variety of single-atom catalysts (Pt, Pd, Rh, Cu, Pb, Bi, and Sn).

Another type of 0D material anchored on ATM substrates in order to construct heterointerface ATM catalysts involves atomic-scale compounds. For instance, Cui and co-workers synthesized atomically dispersed MoO_x_ species anchored on Rh metallene following the interface engineering design principle through a one-pot solvothermal method [35]. The obtained catalyst generates an oxide–metal interface with a large extent of exposure, acting as the active center for alkaline HER. The electron-depleted MoO_x_ sites at the interface favor adsorption and dissociation of H_2_O molecules, while the adjacent Rh atom acts as the active site for H* adsorption and H_2_ generation. The delicately designed heterojunction catalyst delivers an extremely low overpotential of 15 mV at 10 mA·cm^−2^ and a high mass activity of 2.32 A mg_Rh_^−1^ at an overpotential of 50 mV. Furthermore, the large number of electrons accumulated at the interface contributes to the stability of the MoO_x_ species (Figure 2B), leading to durability for 10,000 potential cycles with negligible current density loss.

## 3. Modulation Strategies for Heterointerfacial ATMs’ Electronic Properties

The delicately designed atomically thin interface catalysts enhance catalytic performance by modulating physicochemical characteristics (i.e., activity, selectivity, and stability). Despite the different structural forms of heterointerfacial ATMs, the intrinsic modulation strategies of their electronic properties are almost similar. We classify the modulation strategies into two main effects: the electronic effect and strain effect. The electronic structure is vital to the catalytic performance, as the electron transfer at the interface alters the electronic structure, tuning the Fermi level and work function, and leads to an electronic effect [36]. Moreover, the atomic thickness of ATMs endows them with an intrinsic flexible structure, leading to a strain effect when combined with other materials to form an interface, resulting in a curved morphology and inherent strain within the materials. The strain effect can indirectly modulate the electronic structure of catalysts, which is similar to the electronic effect. In addition, it can modulate the coordination numbers around the strain-generated vacancies. It is worth noting that both the electronic effect and strain effect can enhance the intrinsic catalytic activity, and a well-designed atomically thin interface can maximize these effects. 

### 3.1. Electronic Effect

Heterointerfacial ATMs typically consist of components with different work functions and Fermi levels, leading to spontaneous electronic interaction when in intimate contact. This interaction results in charge/electron redistribution, altering the electronic structure near/within the formed interface [28]. These alterations, together with easy electron transfer, are vital to the optimization of a catalyst’s catalytic performance [37]. We further categorize this specific modulation strategy into two specific effects: direct charge/electron transfer and indirect modulation strategies (i.e., d-band center induced modulation, BIEF/interfacial polarization modulation). The electronic effect can also change the electronic structure via charge redistribution, further enhancing catalytic performance. For instance, by linking the single atomic W with Rh metallene through the oxygen bridge, a number of electrons transfer from the W to the Rh and tune the density of state of Rh active sites [38]. Consequently, the intrinsic activity of the Rh active sites is enhanced, delivering a superior HER performance with an ultrahigh mass activity of 259.19 A g^−1^. Sho et al. fabricated a heterointerface structure by loading an Au cluster on the unilamellar layered double hydroxide nanosheets [39]. The electrons transfer from the Au to the nanosheets, facilitating the formation of active sites on the surface and enhancing the OER activity.

#### 3.1.1. Direct Charge/Electron Transfer Modulation

**Figure 3:**
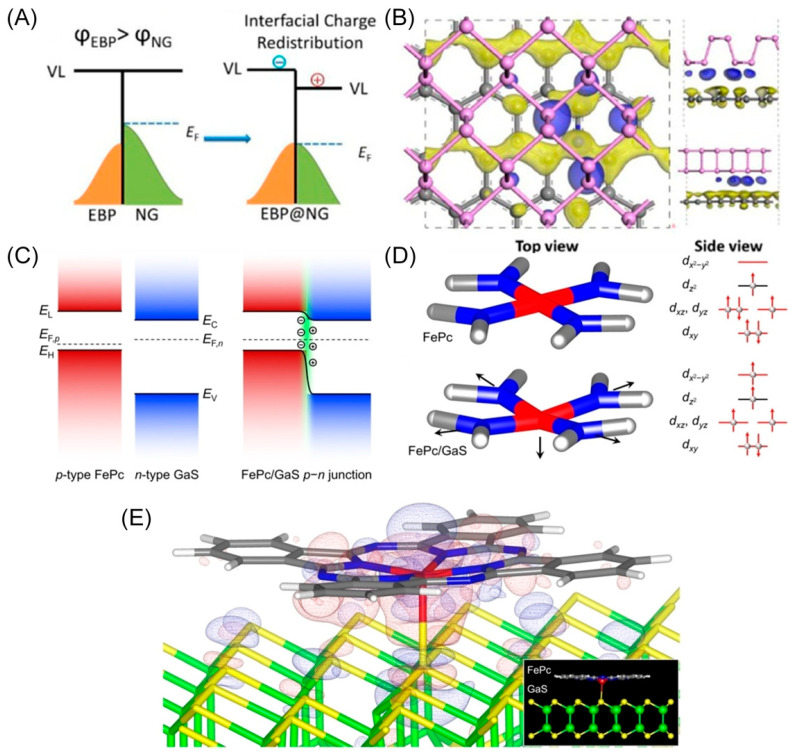
(**A**) Schematic illustration of electron transfer from NG to EBP and interfacial charge redistribution. (**B**) Schematic illustration of the specific charge density difference between NG and EBP, where the blue area represents electron-rich areas and the yellow area represents hole-rich areas. Reproduced with permission: Copyright 2019, American Chemical Society [29]. (**C**) Schematic illustration of electron transfer from GaS to FePc and interfacial charge redistribution. (**D**) The discrepancies of atomic structure (left) and electronic configuration (right) of the FeN_4_ moieties. It shows the distorted FeN_4_ moieties and electronic configuration in FePc/GaS (bottom) compared with pristine FePc (upper). (**E**) Schematic illustration of the charge density difference at the junction interface. The red area represents the electron accumulated area and the blue area represents the electron depletion area. Inset shows a side view of the structurally relaxed model of the junction. Reproduced with permission: Copyright 2022, Wiley-VCH [40].

Discrepancies in the Fermi level and work functions lead to automatic electron transfer from one component to another at the interface until the Fermi level aligns and an equilibrium of work functions is reached. This direct charge/electron transfer at the active sites alters the electronic structure, affecting the adsorption of reactants/intermediates and catalytic reaction kinetics. For instance, a metal-free 2D/2D heterostructure fabricated with exfoliated black phosphorus (EBP) and N-doped graphene (NG) exhibits electron transfer from NG to EBP due to the lower Fermi level of the EBP (Figure 3A), which coincides with X-ray photoelectron spectroscopy (XPS) results [29]. The accumulated electrons on the EBP optimize the H adsorption free energy (Figure 3B), with DFT calculations showing a drastic reduction in Gibbs free energy of hydrogen adsorption to 0.1 eV. Furthermore, the electron-depleted NG possesses abundant positively charged carbon sites for the OER process, with free energy of the rate-determining step intermediates (OOH*) reducing from 0.51 eV for solely NG to 0.43 eV for the heterostructure. As a result, the resultant catalyst acts as a bi-functional catalyst with overpotentials of 191 mV for the HER process and 310 mV for the OER process at the current density of 10 mA cm^−2^. Assembled as an anode and cathode for the water electrolyzer, the cell voltage is as low as 1.54 V at 10 mA cm^−2^, comparable to the Pt/C@RuO_2_ couple (1.60 V).

From the perspective of work function, a rational combination of n-type semiconductor supports and p-type SACs can form a p–n junction, where the work function discrepancy compensates for charge carriers of opposite signs on the two sides. Li and co-workers developed a rectification strategy for tuning SACs by using the work function of different n-type metal chalcogenides (Figure 3C) [40]. They synthesized well-tuned SACs with a p–n junction structure by combining p-type iron phthalocyanine (FePc) and n-type gallium monosulfide (GaS). The discrepancy in work function generates a space-charged region across the junction interface, distorting the FeN_4_ moiety (Figure 3D) and spin-state transition in the Fe^II^ center. DFT calculation verifies the charge transfer from the upper surface of GaS to the Fe^II^ center, tending to carry a high-spin (HS) state (Figure 3E). Furthermore, the kinetic overpotential of the rate-limiting step is the lowest (0.32 V) compared with pristine FePc and intermediate-spin FePc/GaS junctions, demonstrating enhanced intrinsic ORR catalytic activity. The XANES spectra data confirm the electron transfer at the interface. As a result, the well-tuned p–n junction delivers a half-wave potential of ca. 0.895 V and a 2.5-fold higher kinetic current density of 35.7 and 4.7 mA cm^−2^ at 0.85 and 0.90 V for the ORR process compared to pristine FePc. In conclusion, charge/electron transfer can directly alter the electronic structure of materials, optimizing the adsorption of active sites to reactants/intermediates and boosting catalytic reaction kinetics.

#### 3.1.2. d-Band Center Descriptor

The d-band center theory provides a profound understanding of bond formation and reactivity trends in transition metals. The adsorption strength between transition metal sites and reactants/intermediates is highly correlated with the antibonding states relative to the Fermi level. Stronger bonding between reactants/intermediates and active sites can form when the anti-bonding states are at higher energy levels. As the position of the d-band center determines the antibonding state, adjustments to the d-band center can lead to variations in adsorption free energy [41].

**Figure 4:**
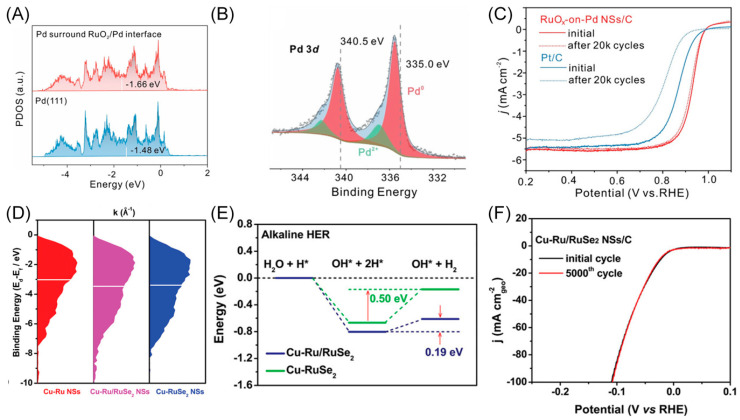
(**A**) PDOS plots showing the downshift of the d-band center of Pd atoms surrounding the RuO_2_/Pd (111) interface (the red one) compared with the ones on a clean Pd (111) surface. (**B**) XPS spectrum of Pd 3d, suggesting that most of the Pd is in a zero-valent state. (**C**) ORR linear sweep voltammetry curves before and after 20,000-cycle accelerated stability tests. Reproduced with permission: Copyright 2023, Wiley-VCH [42]. (**D**) XPS valence-band spectra showing the d-band center downshift of Cu-Ru/RuSe_2_ NSs. (**E**) The DFT results showing the changes in reaction energy in alkaline HER. (**F**) HER linear sweep voltammetry curves before and after 5000-cycle accelerated stability tests of Cu-doped Ru/RuSe_2_ NSs/C in 1.0 M KOH. Reproduced with permission: Copyright 2023, Wiley-VCH [44].

Consequently, many catalysts are designed based on the d-band center theory. For instance, Xie’s group created RuO_x_/Pd interfaces by atomically dispersing RuO_x_ on ultrathin Pd nanosheets (NSs) via a one-pot synthesis method [42]. The DFT calculation results indicate that electrons tend to transfer from the Pt surface to the RuO_x_/Pd interface, accumulating at the interface, as evidenced by the up-shifted binding energy of the Pd 3d orbital in the XPS spectra (Figure 4B). The projected density of states (PDOS) directly reveals the downshifting of the d-band center (Figure 4A), decreasing from the original −1.66 eV of Pd (111) to −1.48 eV after decorating with RuO_x_. As a result of these modulations, the adsorption of the O species is weakened, resulting in the relatively high half-wave potential of 0.93 V. It also exhibits outstanding catalytic activity toward ORR in alkaline media (1.52 A mg_Pd+Ru_^−1^), achieving 22.4 and 8.0 times that of the commercial Pd/C and Pt/C catalysts, respectively. Moreover, the RuO_x_-on-Pd NSs/C demonstrates excellent durability, with only 25.6% mass activity lost after 20,000 cycles (Figure 4C).

Similarly, Lee and co-workers synthesized a Pd-PdO/RuO_2_ atomically thin multicomponent heterostructure using annealing and solution reduction methods [43]. The resultant catalyst modulates the d-band center to a moderate level, optimizing the H* binding strength and increasing the H* adsorption free energy, thus promoting catalytic activities. The interaction between Pd–PdO and amorphous RuO_2_ optimizes the adsorption and desorption of the oxygenated species on the catalytic surface, lowering the energy barrier for potential-determining steps and favoring the reaction energetics. The heterostructure also balances the bonding between the O_2_ and the catalytic surface, resulting in a multifunctional catalyst with excellent performance regarding HER, OER, and ORR.

Furthermore, Guo’s group designed Cu-doped Ru/RuSe_2_ heterointerface nanosheets (Cu-Ru/RuSe_2_ NSs) for highly efficient HER catalysis in alkaline media [44]. The XPS valence-band spectra results confirm the downshifting of the d-band center (Figure 4D). The DFT calculations explain the optimization mechanism, where the binding strength of the proton is weakened, and the energy barrier of H_2_ formation is reduced with the downshifting of the d-band center position. The formed heterointerface nanosheets show a much stronger affinity to H_2_O and a near-zero adsorption free energy for H*. The catalyst also decreases the energy barrier for water dissociation and the rate-determining step (Figure 4E). Correspondingly, the resultant catalyst exhibits a small overpotential of 23 mV at 10 mA cm^−2^, a low Tafel slope of 58.5 mV dec^−1^, and a high turnover frequency value of 0.88 s^−1^ at 100 mV for HER in alkaline media. Furthermore, the interface catalyst demonstrates long-term stability with no apparent activity decay after 5000 cycles (Figure 4F).

#### 3.1.3. BIEF/Interfacial Polarization-Induced Modulation

The generation of BIEF is closely related to charge/electron redistribution during interface formation [45,46]. After the process of electron transfer is finished, the energy difference on each side of the interface induces interfacial polarization, resulting in a potential difference and the formation of BIEF. The electron transfer originating from the generation of BIEF can alter the charge density of active sites and enhance intrinsic activity [47]. Furthermore, BIEF promotes electron transfer at the interface, thus boosting overall reaction kinetics.

**Figure 5:**
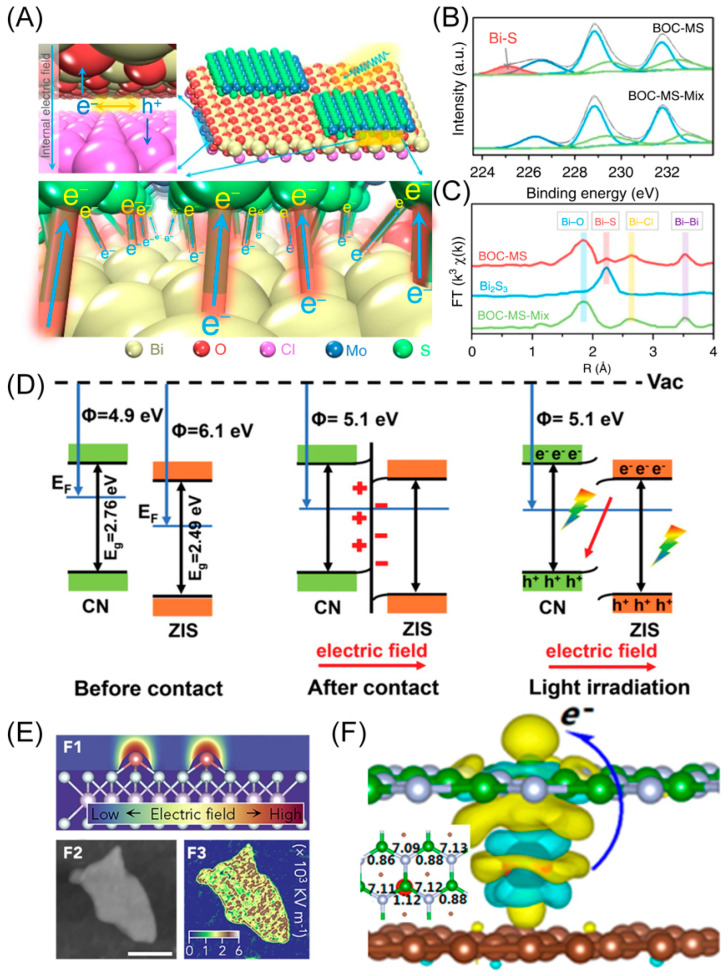
(**A**) Schematic illustration showing the crystal structure of BOC-MS and charge transfer pathway within BOC-MS. The upper left panel illustrates the electron–hole separation process within 1L-BOC. The bottom panel demonstrates the interfacial electron transfer from bottom 1L-BOC to upper 1L-MS along the Bi–S bonds. (**B**) Comparison of XPS spectra between BOC-MS and a physical mixture of BOC and MS (BOC-MS-Mix) showing the electron transfer from the end faces of 1L-BOC to the 1L-MS. (**C**) Comparison of Bi L-edge EXAFS between BOC-MS and BOC-MS-Mix, which validates the existence of Bi–S bonds at the interface. Reproduced with permission: Copyright 2016, Springer Nature [48]. (**D**) Schematic illustration of the proposed charge transfer mechanism. Reproduced with permission: Copyright 2021, Wiley-VCH [51]. (**E**) Computed electric field (up), atomic force microscopy (AFM) images (left), and experimentally measured electric field distribution (right) of SACs-MoS_2_-Fe-2.0. Scale bar represents 50 nm. Reproduced with permission: Copyright 2020, Cell Press [52]. (**F**) Schematic illustration of electron density differences in BN/V/G, which confirms the electron transfer from the Tm atom to the B atom. Reproduced with permission: Copyright 2020, American Chemical Society [53].

For instance, Zhang’s group synthesized a van der Waals-heterostructure catalyst by creating a 2D Janus bilayer junction through the selective and chemical assembly of 1T MoS_2_ monolayers onto (Bi_12_O_17_) the end faces of Bi_12_O_17_Cl_2_ (BOC) monolayers [48]. The internal electric field originating from the BOC can separate the photo-generated electrons and holes, and the separated electrons are transferred to the MoS_2_ (MS) (Figure 5A). The internal electric field directing charge flow within the BOC monolayers is confirmed via mathematical calculations of the electrostatic potential differences between the (Bi_12_O_17_)^2+^ layer and the Cl_2_^2−^ layer. The validation of the Bi-S bond is realized through XPS, extended X-Ray absorption fine structure (EXAFS), and Raman spectra characteristics, providing an interfacial electron transfer pathway (Figure 5B,C). Similarly, the interfacial charge flow along the Bi-S bonds is proven by calculating the electrostatic potentials of different layers. The overall charge flow is also confirmed by photo deposition of Pt and MnO_x_ on the Janus bilayer junction catalyst, where their separated deposition on either side of the junction validates the existence of the hole–electron separation. In summary, the resultant catalyst provides a dual electron transfer pathway among the formed interface, delivering an ultralong carrier lifetime of 3446 ns and a superior visible-light photocatalytic hydrogen evolution rate of 33 mmol h^−1^ g^−1^.

In addition to applications in electrocatalysis, BIEF is also widely applied in photocatalysts, as it can be utilized to regulate the charge transfer direction and lead to a direct Z-scheme photocatalytic mechanism rather than Type II [49,50]. For instance, Bai’s group fabricated an ultrathin 2D ZnIn_2_S_4_/g-C_3_N_4_ Z-scheme heterojunction via the precise in situ growth of ZnIn_2_S_4_ (ZIS) on g-C_3_N_4_ (CN) [51]. According to the electro-statistic potential diagram, the work function discrepancy between ZIS and CN is 1.2 eV, inducing electron transfer from CN to ZIS (Figure 5D). Consequently, BIEF from CN to ZIS is established, enhancing the separation of electrons in the conduction band of ZIS and holes in the valence band of CN. After the interface optimization, the ZIS-100CN delivers a superior photocatalytic performance, with a hydrogen production rate of 14.799 mmol g^−1^ h^−1^, being higher than bare ZIS and CN.

In addition to BIEF, interfacial polarization is emerging as a new modulation strategy, which originates from the combination of SACs with optimal substrates. For instance, Zhang’s group fabricated a superior electrocatalyst for the nitrogen reduction reaction (NRR) by immobilizing protrusion-shaped Fe SACs on MoS_2_ nanosheets [52]. The engendered electric fields induced interfacial polarization which can activate the inert N_2_ and enhance the bond fracture to boost electrocatalytic ammonia synthesis (Figure 5E). Correspondingly, the catalyst delivers a high NH_3_ faradaic efficiency and production rate of ca. 36.1 mmol g^−1^ h^−1^ and 97.5 μg h^−1^ cm^−2^ at −0.2 V (vs. RHE) in 0.1 M KCl.

Furthermore, Jiang’s group developed a new materials design strategy by intercalating a single transition metal atom into hexagonal boron nitride (h-BN) and graphene nanosheets (BN/TM/G) using first-principle calculations [53]. The interfacial polarization drives the electronic charge transfer from the transition metal atom to the B atoms in h-BN, becoming the active sites of N_2_ capture and activation (Figure 5F). The not-strong-not-weak interfacial polarization electric field optimizes the binding strength of the key intermediate *N−NH. The NRR catalytic performance of the BN/TM/G system is highly correlated with the degree of positively polarized charges on the transition metal atom. Consequently, the BN/TM/G with Ti and V as the interfacial single-atom intercalation guest is confirmed to be a promising NRR catalyst with high stability, offering excellent energy efficiency and suppression of the competing hydrogen evolution reaction. 

### 3.2. Strain Effect

The strain effect generally originates from the lattice mismatch when using materials with different lattice parameters or from the curvature morphology during the formation of a core–shell heterostructure. Inducing strain in the atomically thin heterointerfacial catalysts can indirectly modulate their electronic structure. The strain effect leads to alteration in the crystal and electronic structure and modulation in atomic bonding through d-band state alterations, thereby enhancing catalytic activity and effectively modulating the binding strength and adsorption behavior of molecules [54,55,56]. The variation in electronic structure can be verified through experimental characterization using XPS and X-ray absorption fine structure (XAFS) as well as computational analysis with a Bader charge density diagram.

**Figure 6:**
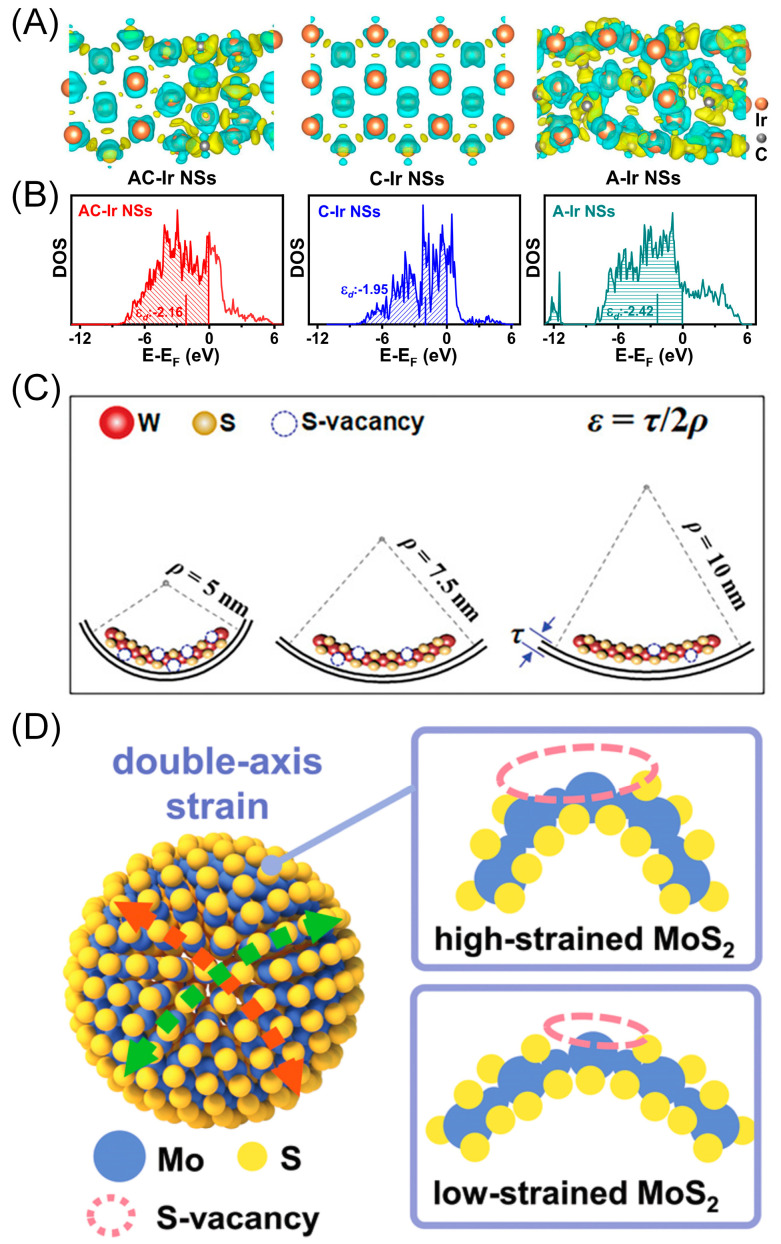
(**A**) The Bader charge density analysis results of AC-Ir NSs with 4% tensile strain (**left**), crystalline-Ir NSs (**middle**) and amorphous-Ir NSs (**right**), respectively. The cyan areas represent the charge depletion areas and the yellow represent the charge accumulation areas. (**B**) The PDOS plots showing the optimized d-band center of AC-Ir NSs with 4% tensile strain (the red one) among the three catalysts. Reproduced with permission: Copyright 2022, Springer Nature [56]. (**C**) Schematic illustration of S-vacancy density difference in WS_2_ nanosheets with various pore sizes. Reproduced with permission: Copyright 2020, Wiley-VCH [57]. (**D**) Schematic illustration of strain-induced S-vacancy generation mechanism in an MoS_2_ model. Reproduced with permission: Copyright 2022, Wiley-VCH [58].

For instance, Hong and co-workers developed a facile method to modulate the planar strain in Ir nanosheets by creating high-density in-plane amorphous–crystalline phase boundaries, leading to about 4% tensile strain in the boundaries [56]. The resultant amorphous–crystalline Ir nanosheets (AC-Ir NSs) possess atomically thin characteristics with a thickness of 5 nm, which benefits the exposure of the strained interface. The in-plane tensile strain induces electron transfer from crystalline domains to amorphous domains in the nanosheets (Figure 6A), downshifting the anti-bonding state of the Ir sites and weakening the Ir–H bond strength (Figure 6B). Therefore, the HER performance of the catalyst is improved, delivering a low overpotential of 21 mV at 10 mA cm^−2^ in an alkaline medium, with a commercial-Pt/C-comparable Tafel slope of 27 mV dec^−1^, as proven by the relatively low hydrogen adsorption Gibbs free energy (−0.06 eV). Moreover, it possesses long-term stability with negligible degradation after 20,000 cycles.

Introducing tensile stain into atomically thin TMDs favors vacancy generation, which can act as HER active sites and activate the inert basal plane for boosting HER. For instance, Dong and co-workers synthesized WS_2_@graphene van der Waals heterostructures through the epitaxy growth method [57]. The electronic coupling between the heterostructure facilitates electron and mass transfer at the interface, enhancing HER catalytic performance. Furthermore, the spherical structure of the graphene substrate generates in-plane strain and S-vacancies in the as-grown WS_2_ nanosheets. The integration of the strain effect and vacancies remarkably decreases the free energy of hydrogen adsorption, thereby facilitating the HER activity of the catalyst. By adjusting the pore size of the graphene substrate, the strain and S-vacancies can be precisely controlled (Figure 6C). Consequently, the synthesized heterostructure requires an overpotential of 117 mV to achieve a current density of 10 mA cm^−2^. Fan’s group synthesized biaxially strained MoS_2_ nanoshells on a single-crystalline Ni_3_S_2_ core, forming a heterostructure with the MoS_2_ layer precisely detained between one and five layers (Figure 6D) [58]. The resultant catalyst exhibits remarkable HER activity with a relatively small overpotential of 78.1 mV at 10 mA cm^−2^ and long-term durability with negligible activity degradation after 2000 cycles. The superior catalytic performance is attributed to the synergistic effect between biaxial strain and induced sulfur vacancies, which decrease the hydrogen adsorption free energy of S sites and further change the potential-determining step with the assistance of S vacancies.

## 4. Application

The ultimate purpose of developing novel design strategies for heterointerfacial ATMs is to significantly enhance overall performance. As mentioned above, the atomic thickness provides a high specific area, facilitating the easy access of reactants/intermediates to active sites. Furthermore, the delicately optimized electronic structure drastically enhances overall catalytic performance. These inherent advantages render heterointerfacial ATMs highly applicable to various energy-related applications. In this section, we comprehensively summarize the applications of atomically thin interface catalysts in energy conversion and storage, encompassing HER, OER, ORR, and CO_2_RR, as well as their applications in photocatalysts and rechargeable batteries.

### 4.1. HER

**Figure 7:**
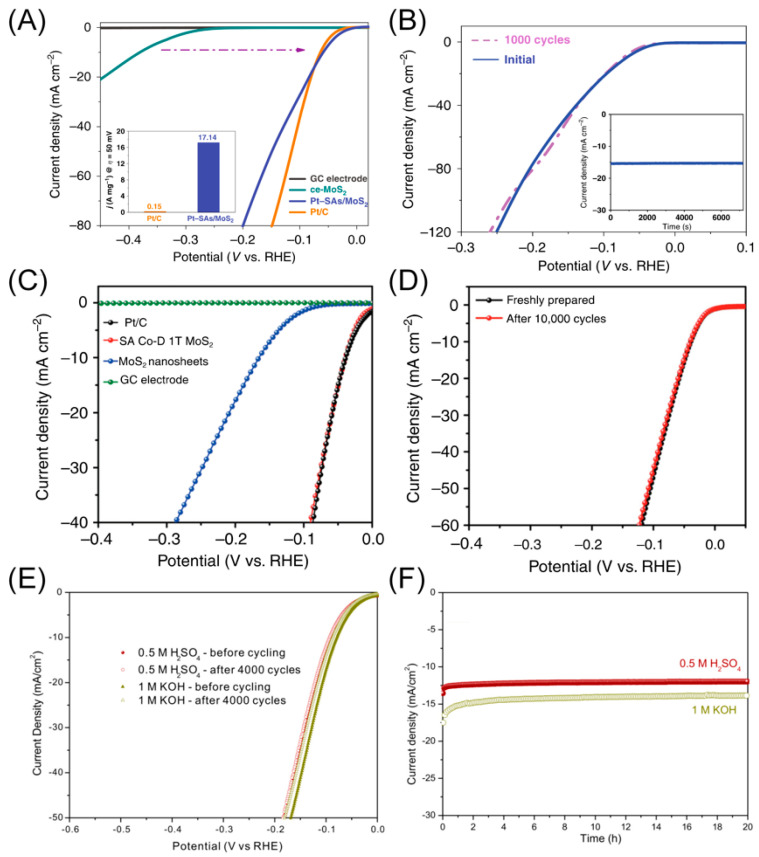
(**A**) HER linear sweep voltammetry curves showing the HER activity of Pt-SAs/MoS_2_-covered glassy carbon electrodes in 0.5 M H_2_SO_4_ solution, which is comparable to that of commercial Pt/C. Inset shows the mass activity of Pt-SAs/MoS_2_ superior to the commercial Pt/C. The dashed arrow illustrates the performance improvement of the MoS_2_ after loading with Pt single atoms. (**B**) Stability test before and after 1000 cycles of Pt-SAs/MoS_2_ through potential cycling. The inset time-dependent current density curve of Pt-SAs/MoS_2_ also shows a negligible current density decrease after running for 2 h in 0.5 M H_2_SO_4_ solution at a constant overpotential of *η*  =  80 mV. Reproduced with permission: Copyright 2020, Springer Nature [34]. (**C**) HER linear sweep voltammetry of different catalysts obtained in Ar-saturated 0.5 M H_2_SO_4_. (**D**) Polarization curves of the SA Co-D 1T MoS_2_ showing negligible current density loss after 10,000 cycles. Reproduced with permission: Copyright 2019, Springer Nature [60]. (**E**) Stability test and (**F**) time-dependent current density curve of HER under both acidic and alkaline conditions at an overpotential of 100 mV. Reproduced with permission: Copyright 2018, American Chemical Society [61].

Water electrolysis, or overall water splitting, represents a practical method for large-scale hydrogen generation. Hydrogen, known for its clean and renewable attributes, offers high energy density, and its combustion products without any CO_2_ or NO*_x_* pose no harm to the environment [59]. Furthermore, to replace the fossil-fuel-based energy structure with carbon-neutral and environmentally friendly hydrogen energy, the development of effective catalysts for the HER process is crucial. For instance, atomically thin MoS_2_ nanosheets with single dispersed Pt atoms deposited on them have been fabricated and proved to be exceptional catalysts for HER [34]. The resultant Pt-SAs/MoS_2_ exhibits excellent electrocatalytic activity, requiring only a negligible overpotential of ca. 59 mV at 10 mA cm^−2^ (Figure 7A). Additionally, it displays a high mass activity of 17.14 A mg^−1^ at an overpotential of 50 mV, a small Tafel slope of 31 mV dec^−1^, an extremely high exchange current density of 2.24 mA cm^−2^, and remarkable long-term stability without a significant decline in current density after 1000 cyclic voltammetry cycles or 2 h of running (Figure 7B). The outstanding catalytic activity is mainly attributed to the redistribution of the electronic structure, with electrons automatically transferring from the single-atom Pt to the MoS_2_ nanosheets. Furthermore, the hybridization between Pt (5d orbitals) and neighboring S atoms effectively enhances the dominance of d-electron orbitals near the Fermi level, improving the catalytic activity and electronic energy levels for better conductivity. The free energy for hydrogen adsorption of the as-prepared catalyst is −0.067 eV, representing more favorable HER behavior than the commercial Pt catalyst (−0.139 eV).

Zheng and co-workers produced a single-atom cobalt (Co) array covalently bound to distorted 1T MoS_2_ nanosheets (SA Co-D 1T MoS_2_) [60]. The catalyst presents high electrocatalytic activity for HER in an acidic solution, with a small onset overpotential of 42 mV, a Tafel slope similar to Pt/C (32 mV dec^−1^), and excellent long-term durability with a negligible decline in current density after 10,000 cycles and 10 days’ reaction time (Figure 7C,D). The remarkable HER catalytic performance is attributed to the ensemble effect between the MoS_2_ support and the Co single atom, as the empty state of Co close to the Fermi level increased, promoting hybridization between the Co adatom and hydrogen atoms and facilitating hydrogen adsorption on cobalt. 

Moreover, Cai and co-workers fabricated atomically thin Mo/Mo_2_C heteronanosheets, delivering low overpotentials of 89 mV at 10 mA cm^−2^ in acidic solution and 79 mV at 10 mA cm^−2^ in alkaline solution, with high durability and negligible overpotential decay over 4000 sweeps (Figure 7E,F) [61]. The formation of a double-phase interface provides an electron-transfer channel, leading to modifications in the electronic structure by transferring electrons to the β-Mo_2_C and increasing the electron density around the Fermi level of β-Mo2C. These modulations weaken the Mo-H bond strength and decrease the hydrogen adsorption free energy to a thermal-neutral level (−0.04 eV).

### 4.2. OER

Since the sluggish reaction kinetics of the four-electron process and the high onset potential (1.23 V) largely determine the overall water splitting reaction rate [62,63], the steps that are considered to hold back the OER reaction process are water dissociation and oxygen formation. While noble metals like Ir and Ru exhibit superior catalytic activity, their scarcity and high cost limit their practical application [23]. Therefore, there is significant interest in developing OER catalysts with inexpensive and feasible raw materials that can deliver a favorable catalytic activity comparable to noble metal catalysts. 

**Figure 8:**
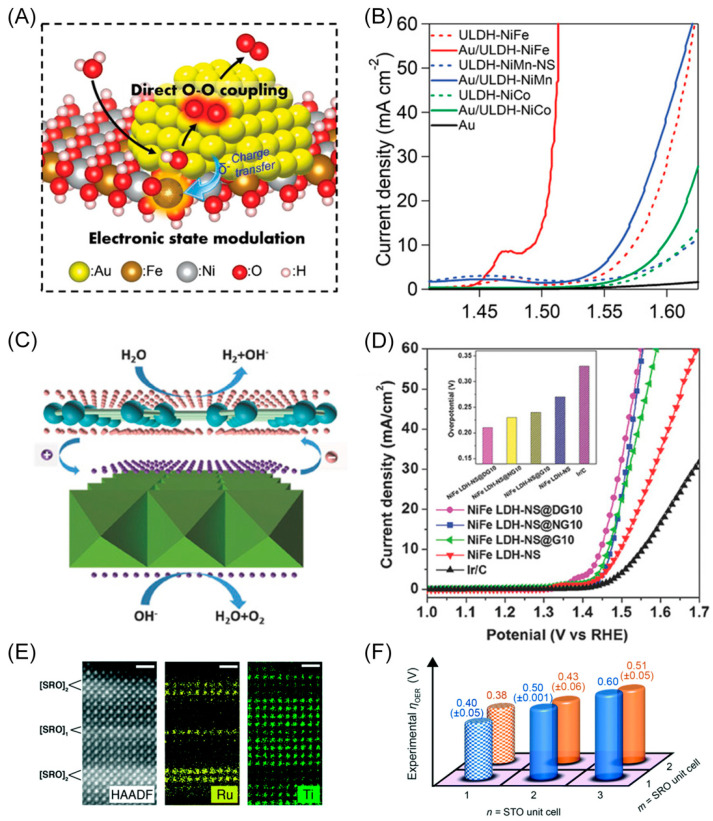
(**A**) The schematic illustration showing the positions of active sites at the interface between the Au clusters and LDHs. (**B**) Linear sweep voltammetry curves showing the better OER performance of Au/LDHs (solid line) than LDHs (broken line). Reproduced with permission: Copyright 2022, Wiley-VCH [39]. (**C**) The schematic illustration of the probable electrocatalytic mechanism. The accumulated electrons (the pink ones) on DG boost HER activity and the accumulated holes (the purple ones) on Ni–Fe LDH facilitate OER activity. (**D**) OER polarization curves of different electrocatalysts in 1.0 M KOH. Inset: the overpotential of different electrocatalysts at a current density of 10 mA cm^−2^. Reproduced with permission: Copyright 2017, Wiley-VCH [64]. (**E**) High-angle annular dark-field imaging (HAADF), scanning transmission electron microscopy (STEM) imaging, and Ti and Ru energy dispersive X-ray analysis (EDX) maps showing different [SRO]*_m_* (*m* = 1, 2) layer positions sandwiched between [STO]_4_ [16,66] layers. Chemical element maps are given for Sr, Ti, and Ru. The scale bar is 1 nm. (**F**) Required overpotentials of different [STO]*_n_*[SRO]*_m_* heterostructures at a current density of 2 μA cm^−2^. Reproduced with permission: Copyright 2018, Royal Society of Chemistry [65].

Yamauchi and co-workers fabricated a heterointerface by loading Au clusters on atomically thin unilamellar exfoliated Ni–Fe layered double hydroxide (Ni–Fe LDH). Their catalyst delivers an excellent OER catalytic performance with an overpotential of 189 mV at 10 mA cm^−2^ (Figure 8B) [39]. This striking activity is ascribed to the charge transfer from the Au clusters to the LDH (Figure 8A), modifying the oxidation states of the metal ions and making them active sites. Furthermore, the delicately designed heterointerface also develops a new pathway for the OER, involving direct interfacial O-O coupling. The synergistic effects of these two mechanisms significantly enhance the intrinsic electrocatalytic activity towards OER.

Yao’s group built a heterostructure by coupling exfoliated Ni–Fe LDH nanosheets and defective graphene (DG) [64]. According to the DFT calculations, charges transfer from the Ni–Fe LDH to graphene and accumulate at the defect sites in graphene after composing the heterostructure (Figure 8C). The electron-deficient Ni–Fe LDH improves the OER performance, while the high defect density of the DG boosts active sites in the resultant heterostructure. Consequently, the assembled catalyst exhibits high electrocatalytic activity for OER in an alkaline solution, with an overpotential of 210 mV at a current density of 10 mA cm^−2^ (Figure 8D). Furthermore, the accumulated electrons at the defect sites also facilitate the HER process, delivering an overpotential of 115 mV at a current density of 20 mA cm^−2^. When applied as a bifunctional catalyst electrode for overall water splitting, the device can deliver a current density of 20 mA cm^−2^ with a voltage of only 1.5 V. In conclusion, the synergetic effects of the highly exposed 3d transition metal atoms of LDH nanosheets and carbon defects of graphene are essential for the bifunctional activity of OER and HER.

Additionally, by embedding only one unit cell of SrRuO_3_ (SRO) beneath the SrTiO_3_ (STO) substrate through the atomically precise heteroepitaxial deposition method (Figure 8E), spatially accessible electronic states are introduced in/near the band gap of STO, changing the electronic structure and elevating the highest occupied state (HOS) from the valence band maximum to the Fermi level [65]. This elevation of HOS results in increased charge transfer from the HOS to the O 2p adsorbate state during surface adsorption of O, leading to a more stabilized adsorbate–surface bond and enhanced surface activity. Consequently, the activity of STO toward OER is activated, while the excellent stability is retained, forming a robust OER catalyst. Furthermore, embedding only two unit cells of SRO beneath the STO substrate prevents the dissolution of SRO while preserving the OER activity (Figure 8F). DFT results demonstrate that the combination of two unit cells of STO and one unit cell of SRO beneath the STO capping layer strikes a balance between activity and stability, as the number of states at the Fermi level is appropriate.

### 4.3. ORR

ORR occurs at the cathodes in energy conversion/storage systems such as fuel cells and metal−air batteries. The sluggish kinetics of ORR pose a significant bottleneck for these energy devices, impeding the ideal four-electron transfer process and requiring a high overpotential to drive the reaction at a reasonable rate. Therefore, extensive efforts are focused on exploring new ORR cathode catalysts [16,66]. Pt-based catalysts are state-of-the-art catalysts among all ORR catalysts. By fabricating the Pt atomic layers through the converting of single-layer PtO_x_, the usage of Pt has been reduced while maintaining the ORR catalytic activity [67]. The high electrochemically active surface area (124 m^2^ g^−1^) endows the resultant Pt nanosheets with excellent activity, with a high mass activity of 415 A g^−1^ and a specific activity of 0.330 mA cm^−2^. Durability is another vital evaluation index for ORR catalysts. Using graphene as a growth template, the atomically thin 2D Pt layer was grown on it [68]. In addition to providing a growth platform, the graphene also prohibits the dissolution or agglomeration of the Pt layer. As a consequence, this architecture delivers an ORR performance comparable to commercial Pt/C catalysts and superior durability with no activity loss in 1000 ORR cycles.

### 4.4. CO_2_RR

The development of CO_2_ reduction technology is promising as it can simultaneously mitigate the greenhouse effect and produce organic fuels to tackle the fossil fuel dilemma [23]. However, the activation of CO_2_ and the selectivity of products remain significant challenges in CO_2_RR. Although the Earth-abundant metal copper is a low-cost element, its high overpotential when utilized as a catalyst still needs to be solved [15]. Hence, exploiting inexpensive catalysts with high activity and selectivity is desirable.

Zhou’s group built a photocatalyst by implanting single Cu atoms on Ti_0.91_O_2_ atomically thin single layers [69]. The deposition of Cu promotes the formation of neighboring oxygen vacancies in the Ti_0.91_O_2_ matrix, leading to high electron-based selectivity of 64.8% for C_3_H_8_ (product-based selectivity of 32.4%) and 86.2% for total C_2+_ hydrocarbons (product-based selectivity of 50.2%). The enhanced photocatalytic performance and selectivity are attributed to the thermodynamically favorable C_1_-C_1_ and C_1_-C_2_ coupling process, along with a decreased free energy of the potential-determining step for the C_3_H_8_ formation pathway. These improvements stem from the electronic and geometric effects of the single-atom Cu-induced oxygen vacancies. Furthermore, the stabilized key *CHOCO and *CH_2_OCOCO intermediates effectively suppress the formation of CO. 

In a separate study, Zhang’s group fabricated a 2D van der Waals heterostructure on graphene to achieve high-efficiency CO_2_ electroreduction, where a metalloporphyrin covalent organic framework (Co-u-COF) was epitaxially grown on graphene [70]. The strong interlayer coupling leads to electron-deficient metal centers, accelerating the electrocatalysis process. According to the DFT calculation results, the discrepancy in work function between the two layers induces a substantial charge transfer at the layer interface, depleting the charge originally accumulated at the Co-N_4_ site. This interface charge transfer increases the ability of Co-N_4_ for *COOH adsorption, as confirmed by the XPS and XANES characteristic results. Furthermore, the free energy of the CO_2_-to-*COOH transformation decreases to 0.05 eV, altering the rate-determining step from the first electron transfer to the desorption of CO. In conclusion, the incorporation of graphene considerably changes the catalytic kinetics of CO_2_RR on Co-u-COF, exhibiting a CO faradaic efficiency of 97% at a partial current density of 8.2 mA cm^−2^ in an H-cell with a durability lasting for over 30 h. The selectivity of CO could approach 99% with a partial current density of 191 mA cm^−2^ in a liquid flow cell, surpassing most reported organometallic frameworks.

### 4.5. Applications in Photocatalysts

The direct utilization of feasible and inexhaustible solar energy is highly commendable. Given the shared design principles between electrocatalysts and photocatalysts, the above-mentioned atomically thin heterointerface modulation strategies can also be extended to photocatalysts.

A Z-scheme heterojunction was fabricated by loading Co phthalocyanine (CoPc) on a 2D B-doped and N-deficient g-C_3_N_4_ (P-BNDCN) substrate [71]. The B dopants and N defects simultaneously tune the conduction and valance band locations of g-C_3_N_4_, enhancing the absorption ability of the catalyst with regard to visible light. Meanwhile, the introduction of CoPc provides abundant single active sites and further improves the light absorption of the P-BNDCN. Consequently, this configuration facilitates the cascade charge transfer through the fabricated Z-scheme heterojunction, further enhancing the intrinsic catalytic performance. The resulting CoPc/P-BNDCN exhibits excellent photocatalytic CO_2_ reduction activity, with a generation rate of 197.76 and 130.32 μmol h^−1^ g^−1^ for CO and CH_4_, respectively.

### 4.6. Applications in Catalyst-Based Rechargeable Batteries

The urgent development of rechargeable batteries aims to maximize electronic power storage within limited battery volumes [72]. Lithium-ion batteries (LIBs) are state-of-the-art rechargeable energy storage devices widely applied in portable electronic devices and electric vehicles. Meanwhile, various rechargeable batteries, such as zinc–air batteries and sodium/potassium-ion batteries, are being developed. The application of heterointerfacial ATMs in rechargeable batteries is extensively studied due to their high specific surface area and ultrathin thickness, which can increase capacity and reduce battery volume.

For instance, Zhao’s group synthesized vertically stacked ultrathin 2D single-layered ordered-mesoporous-carbon/2D MoS_2_ layered heterostructures through the polymerization and carbonization of single-layered monomicelles [73]. When applied as an anode for LIBs, the atomically clean and well-defined interface between the MoS_2_ and the mesoporous-carbon layer provides a shortcut for fast electron transport, while the abundant and accessible 2D ordered mesopores build a shortened Li^+^ diffusion pathway. As a result, the electrode delivers highly reversible capacities of 1400 mA h g^−1^ at 100 mA g^−1^ and retains ca. 400 mA h g^−1^ at an ultrahigh current density of 10 A g^−1^, with excellent cycling stability (over 300 cycles). Wu’s group synthesized cathode materials for zinc-ion batteries by forming a 2D heterostructure of ultrathin amorphous vanadium pentoxide uniformly grown on graphene [74]. The unique heterostructure design facilitates fast ion diffusion, and the abundant active sites originating from the amorphous feature and high electrical conductivity of graphene result in a highly reversible aqueous ZIB with an ultrahigh capacity of 489 mA h g^−1^ at 0.1 A g^−1^ and an admirable rate capability of 123 mA h g^−1^ even at 70 A g^−1^. Furthermore, when assembled into a new-concept prototype planar miniaturized zinc-ion micro battery, it demonstrates a high volumetric capacity of 20 mA h cm^−3^ at 1 mA cm^−2^, and maintains long cyclability with high-capacity retention of 80% after 3500 cycles.

## 5. Perspectives

In the past few years, heterointerfacial ATMs have garnered significant attention due to their favorable structural and electronic properties, including an atomic thickness and an interfacial effect originating from the unique structural construction and design principles. These materials have been extensively studied, making them promising candidates for various applications. This review focuses on recent advances in heterointerfacial ATMs, including design principles, modulation strategies, and applications. Although heterointerfacial ATMs are vital in electrocatalysis and have drawn significant research interest, several challenging yet promising aspects still require enormous efforts for further development.

1. Exploration of new material design strategies

Fabricating single-atom vacancies constitutes an atomic-scale modification to regulate the surface of materials, akin to SACs. In contrast to creating protrusion, the formation of vacancies digs down beneath the surface. As SACs can generate localized interfaces when assembled with atomically thin substrates, vacancies could be recognized as localized interfaces in the absence of single atoms. However, further investigation is required to fully comprehend this type of interface effect.

2. Development of novel material synthesis methods

To fully exploit the potential of heterointerfacial ATMs, the utilization of effective approaches for delicate design and precise structure control is vital for catalytic performance modulation. Within this context, various synthesis methods have been exploited to achieve an ultrathin atomic thickness (e.g., single layer thickness) and a large lateral size. However, achieving atomic accuracy to precisely modulate the electronic structure of active sites comes with a substantial cost in material synthesis, rending it impractical for immediate application. Therefore, future work should explore new preparation methods with a facile synthesis process, controllable cost, and a striking modulation effect on the catalytic performance.

3. Structure analysis with advanced characteristic techniques

To better understand the correlation between the structural construction and catalytic properties, and to precisely identify the interface between the components, effective characterization techniques, e.g., scanning tunneling microscopy (STM) and STEM, XAFS, in situ Raman spectroscopy, in situ Fourier transform infrared spectroscopy (FTIR), and in situ transmission electron microscopy (TEM), are urgently needed. By leveraging advanced characterization technologies, accurate structure identification and a comprehensive understanding of the structure–performance correlation can be achieved.

4. Mechanism exploration with first principle calculations

First principle calculations, such as DFT, offer the capability to simulate the chemical adsorption and desorption energy of reactants/intermediates on active sites, providing a complete interpretation of the fundamental reaction mechanism that complements experimental results. The DFT calculations enable a deep understanding of the impact of catalyst structure on the intrinsic catalytic mechanism. Furthermore, the literature on using DFT as a material design method is emerging, allowing for the prediction of the performance of as-synthesized catalysts. By employing DFT calculations, development costs can be significantly reduced, as it helps to screen out potential failed solutions.

5. Practical applications in the industry

The primary objective of developing novel materials is their practical application. In industrial electrocatalysts, high efficiency and long-term stability remain fundamental criteria. For materials in rechargeable batteries, the key requirements are a high-volume capacity and long-term cycling.

In summary, atomically thin heterointerfacial catalyst development is still in its infancy, garnering tremendous attention and demonstrating a wide range of potential applications. Leveraging evolving advanced characterization techniques and DFT calculations will aid in identifying active sites and interpreting intrinsic mechanisms, thus guiding the design of novel heterointerfacial ATMs and accelerating their widespread adoption in modern society.
